# Community Volunteer Support for Families With Young Children: Protocol for the Volunteer Family Connect Randomized Controlled Trial

**DOI:** 10.2196/10000

**Published:** 2018-07-16

**Authors:** Rebekah Grace, Lynn Kemp, Jacqueline Barnes, Emma Elcombe, Jennifer Knight, Kelly Baird, Vana Webster, Fiona Byrne

**Affiliations:** ^1^ Department of Educational Studies Faculty of Human Sciences Macquarie University North Ryde Australia; ^2^ Translational Research and Social Innovation School of Nursing and Midwifery Western Sydney University Liverpool Australia; ^3^ Institute for the Study of Children, Families and Social Issues Department of Psychological Sciences Birkbeck, University of London London United Kingdom

**Keywords:** randomized controlled trial, volunteer home visiting, families, support services

## Abstract

**Background:**

Use of community volunteers to support vulnerable families is a widely employed strategy with a long history. However, there has been minimal formal scientific investigation into the effectiveness of volunteer home visiting programs for families. There is also a need for research examining whether volunteer home visiting leads to improved outcomes for volunteers.

**Objective:**

The objective of this paper is to describe the research protocol for a pragmatic randomized controlled trial (RCT) of the Volunteer Family Connect intervention, a volunteer home visiting program designed to support families of young children who experience social isolation or a lack of parenting confidence and skills. The project is being conducted in partnership with 3 leading not-for-profit organizations, designed to contribute to the body of evidence that informs decisions about appropriate family support services according to the level of need. It is the first study to examine outcomes for both the families and the volunteers who deliver the service.

**Methods:**

The RCT is being conducted in 7 sites across Australia. We aim to recruit 300 families to the study: 150 control (services as usual) and 150 intervention (services as usual + volunteer home visiting) families. Intervention families will receive the service for 3-12 months according to their needs, and all participants will complete 6 data collection points over 15 months. A minimum of 80 volunteers will also be recruited, along with a matched community comparison group. The volunteers will complete 3 data collection points over 12 months. Primary outcomes include community connectedness and parenting competence. Secondary outcomes include parent physical and mental health; general parent well-being; parent empowerment; the child-parent relationship; sustainability of family routines; child immunization; child nutrition or breastfeeding; number of accidental injury reports; and volunteer health, well-being, and community connectedness.

**Results:**

This effectiveness trial was funded in 2016, and we aim to complete data collection by the end of 2018. The first results are expected to be submitted early in 2019.

**Conclusions:**

There is a need to rigorously assess volunteer home visiting and whether it has a unique and important role on the service landscape, complementary to professional services. This research is the first trial of a volunteer home visiting program to be conducted in Australia and one of the largest of its kind worldwide.

**Trial Registration:**

Australian New Zealand Clinical Trial Registry ACTRN12616000396426; https://www.anzctr.org.au/Trial/Registration/TrialReview.aspx?id=370304 (Archived by WebCite athttp://www.webcitation.org/70q42fU7V)

**Registered Report Identifier:**

RR1-10.2196/1000

## Introduction

Volunteer home visiting is a widely adopted community-based approach to support families, linking vulnerable and isolated families to trained volunteers from their local communities who have experience in parenting or caring for children. Volunteer home visiting programs can take different forms, with many seeking to support families by helping them strengthen their social and community networks, providing families with connections to appropriate local health, welfare, and education services and support information [1]. Volunteers may also work with parents to increase their parenting confidence, encourage positive parent-child relationships, share local knowledge, and foster a sense of belonging and community resilience [2]. Bronfenbrenner’s bioecological model [3,4] emphasizes that building resilience in both parents and the communities in which they live is critical to achieving family physical, mental, and social well-being. Bronfenbrenner described a complex and dynamic web of relationships that exist between children, their families, the settings in which children participate, and the wider community. Child health and well-being outcomes are strongly influenced by the many social and environmental contexts that operate within a child’s life. Factors across contextual layers accumulate to increase a child’s or a parent’s resilience or risk factors. This requires the development of social infrastructure to support the growth of inclusion networks and opportunities for meaningful civic participation [1].

While previous research has demonstrated that a sense of belonging and inclusion in the local community context is fundamental to health and well-being [5,6], there are increasing reports of isolation, segregation, and nonparticipation in response to changing community environments [7]. A sense of isolation is particularly evident in research examining the social inclusion of families in need of additional supports, such as new arrivals to a country [8], those with demanding care responsibilities [9,10], and those who experience cognitive limitations or mental health challenges [11].

There is an argument for volunteer home visiting having a unique and necessary place on the landscape of services available to families because of the following reasons: (1) It fills the service gap for families whose circumstances do not meet the eligibility criteria for targeted or sustained professional home visiting services and yet need more support than is available from universal primary health and community services and (2) It is designed to break down potential barriers to service access, such as language, transport, or cultural barriers. Another unique feature of the volunteer home visiting model is that there are two groups within the community who, according to emerging evidence, potentially benefit—the families who receive the service and the volunteers who deliver the service [12-14].

Despite its long history and critical role within Australian service systems, there has been relatively little formal scientific investigation into the effectiveness of volunteer home visiting programs. Comprehensive reviews criticize the available evidence for volunteer home visiting as being largely characterized by research with methodological limitations that is focused on program satisfaction and experiences of participation rather than outcomes [15,1]. Nonetheless, findings from the existing literature suggest there is a role for volunteer home visiting in supporting families with vulnerabilities. International research indicates that this service model may provide an acceptable vehicle for the distribution of health and parenting information [16] and improve family social support networks, both in terms of social capital as well as family social connectedness [17,18]. It has also been shown that volunteer support can contribute to improved outcomes relating to parental emotional well-being [19,20], parental sense of competence [21-23], parent-child relationships [24], and parenting behaviors and skills [25]. There is particularly strong evidence that peer support can play a key role in promoting increased rates of breastfeeding and child immunization [26-28]. There is also potential for volunteer home visiting models to play an important support role in the care plans of those with chronic health conditions [29]. It should be noted, however, that community volunteers may not have a marked impact on clinical outcomes, which may be more appropriately addressed by professional services [30], and volunteer support needs to be provided within the context of well-developed guidance and supervision [31,32]. The small number of studies that examine volunteering in the context of family support programs suggest that volunteers experience positive outcomes such as increased knowledge and skills, a stronger sense of social cohesion, reduced loneliness and isolation, and an improved sense of purpose and confidence [11].

The aim of this research is to rigorously explore the effectiveness of the Volunteer Family Connect program (ACTRN12616000396426), a volunteering home visiting program collaboratively designed by a consortium of researchers and service providers in Australia to support families of young children who are vulnerable because they experience social isolation or a lack of parenting confidence and skills. Volunteer Family Connect is a community-based strategy that aims to improve the well-being, social connection, and parenting of vulnerable families with young children and the well-being and social connection of community members who volunteer. The results can be used to inform public policy on this issue.

## Methods

### Study Design

A pragmatic randomized trial design is being undertaken to provide high-quality evidence to assess the impact of the Volunteer Family Connect program. Pragmatic trials are a rigorous method for assessing effectiveness, that is, the degree of beneficial effect of intervention programs in real-world conditions, answering the question “Does this intervention work under usual conditions?” [30]. In keeping with the “real-world” conditions for a pragmatic randomized trial, in this study, we performed the following:

- We recruited the full range of families referred to the volunteer home visiting programs of the partner organizations through usual referral processes, with no changes to service inclusion and exclusion criteria.
- We compared the volunteer home visiting program with other usual care support services, such as group activities and referral to other agencies.
- We tested real-world implementation of the volunteer home visiting program by our service partners with their current volunteer providers using guidelines to support quality service provision, but acknowledging that there are variations in practice, while rigorously assessing outcomes using standardized measurement tools.

**Figure 1:**
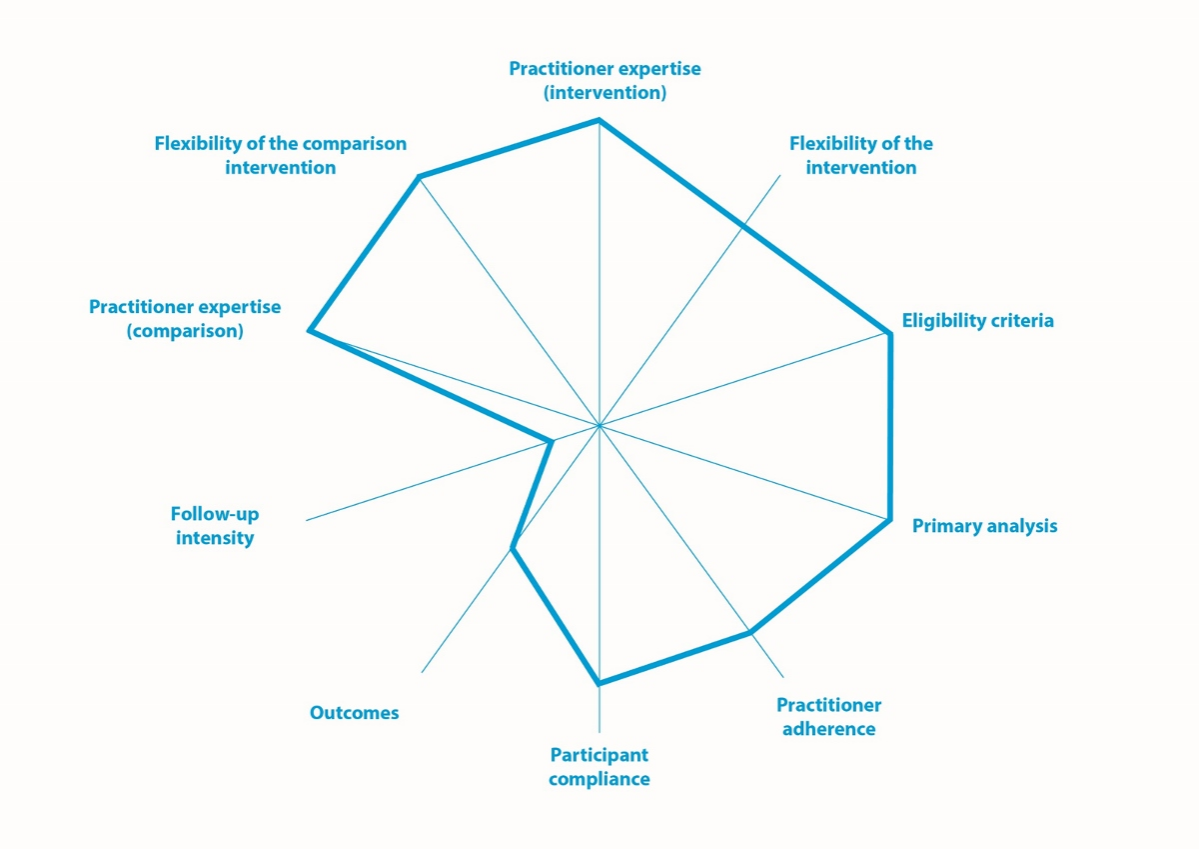
Using the PRagmatic Explanatory Continuum Indicator Summary tool to describe the Volunteer Family Connect randomized controlled trial.

The design of the study was supported using the PRagmatic Explanatory Continuum Indicator Summary (PRECIS) tool [33], which assesses the varying degrees of pragmatic (effectiveness) and explanatory (efficacy) trial approaches. Wider webs represent more pragmatic trials: narrow webs represent more explanatory trials. The PRECIS web for the current trial is depicted in Figure 1. Rating of the Volunteer Family Connect trial on the PRECIS tool was completed collaboratively by the research team. All senior members of the research team gathered in a face-to-face meeting and discussed the project as it is reflected in scores on the PRECIS tool until consensus was achieved.

### Primary Research Question

Is a volunteer home visiting service intervention effective in improving the parenting competence and community connectedness of vulnerable families with young children compared with families who receive usual care services in the community?

### Hypothesis

Families receiving a volunteer home visiting service intervention will have significantly better family outcomes at 15 months post program entry (higher sense of parenting competence and stronger community support networks) than those allocated to continue to receive usual community-based support services.

### Secondary Research Questions

1. Do differences exist in the patterns of parent health, well-being, and empowerment and the sustainability of family routines over time between those who receive the Volunteer Family Connect program and those in the services as usual control group?
2. Does volunteer home visiting lead to differing outcomes for children aged 0-5 years in intervention families compared with those in control group families on measures of immunization, breastfeeding duration, nutrition, and accidental injury?
3. Do different patterns of outcomes for intervention families depend on the location (ie, availability and accessibility of health, welfare, and early childhood services in the local area) and the duration of the program (ranging from 3 to 12 months)?
4. Does volunteering on the Volunteer Family Connect program lead to differing outcomes on measures of well-being, health, community connectedness, and self-efficacy for volunteers over time compared with a matched community comparison group?

### Participants

#### Eligibility Criteria

Eligible families will be those who meet the following criteria: (1) families having one or more children in the 0-5 age range; (2) families at risk of geographic or social isolation (eg, separated from usual support networks due to immigration); (3) parents seeking to develop confidence and increase their parenting knowledge and skills; (4) families residing in the specified service area; and (5) families unable to resource or access other support services (eg, due to financial hardship). Language translation services have been secured so that families with a first language other than English will not be excluded from participating in the research.

Families will be ineligible for the study if any of the following conditions apply: (1) there is active abuse or domestic violence within the family; (2) there is unmanaged mental illness within the family; (3) substance abuse is an issue within the family; (4) the family is living in an environment unsafe for the volunteer to visit; and (5) the family is under child protection orders or there are unsettled parenting arrangements. Families referred for volunteer home visiting will be assessed for eligibility by the local Volunteer Family Connect program coordinator according to the usual practice, and referrals will be made to other services within the community if the family is ineligible.

All current volunteers within the Volunteer Family Connect program will be invited to participate in the study. It is not possible to examine outcomes for volunteers employing a randomized controlled trial (RCT) design because this would halve the number of volunteers available and significantly impact program implementation. Instead, a community comparison sample will be recruited that will be matched on age, gender, education and employment levels, and geographical location.

#### Recruitment

Family participants will largely be identified through the Volunteer Family Connect usual service referral networks, which include child and family health nurses, general practitioners, or family support workers. The Volunteer Family Connect program is advertised within the community, and parents are welcome to self-refer to the program. If eligible for the program, families will be invited to speak to a member of the research team and, if interested, informed consent for the research will be secured. The family will then be randomly allocated by the research manager using computer-generated random numbers to receive either the volunteer home visiting program in addition to usual care services (Intervention group = Volunteer Family Connect + usual care services) or the usual care services only (Control group = usual care services).

**Figure 2:**
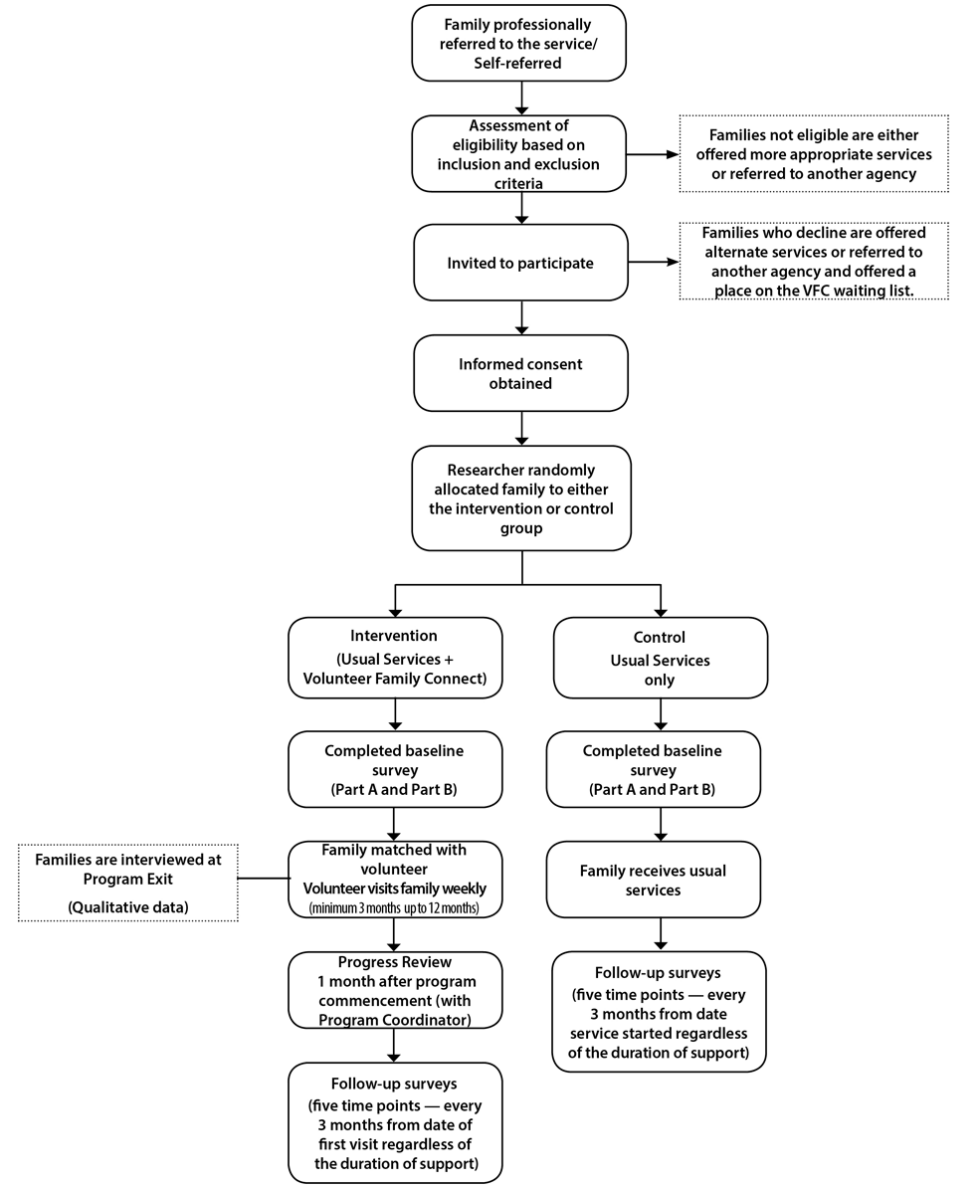
Participant flow diagram.

The procedure used to recruit and allocate families is summarized in Figure 2.

An invitation will be extended to all volunteers currently involved with the 7 participating sites to participate in the research. The matched community comparison sample will be recruited via one of the following two strategies: (1) volunteers will be asked to pass on an invitation to participate in the research to nonvolunteering acquaintances in their local networks and (2) the research will be advertised through Facebook, targeting the local areas in which the Volunteer Family Connect program is being trialed.

#### Sample Size

We aim to recruit 300 families to the study, 150 to the intervention group (Volunteer Family Connect + usual services) and 150 families to the control group (usual services). Recruitment of 150 families per group has been undertaken based on what is feasible given current caseloads in the participating sites and also so that, allowing for attrition, data analysis can be conducted with a final sample size of 100 families per group. A sample size of 100 families per group has power of .80 at the 95% level to detect effect sizes (ESs) of .5 or larger for the PSCS Satisfaction subscale (significant differences detected with minimum n=16 per group) and the Client Enablement Index (significant differences detected with minimum n=7 per group) based on pilot study findings and a previous trial of nurse home visiting conducted by one of the chief investigators on this study [34].

The families will participate in data collection for a period of 15 months. Strategies have been put in place to support and encourage the retention of participants, including asking all participating parents to provide the name and phone number of a relative or friend who can be contacted by the researchers if we struggle to reach them; providing families with the contact details of the research team and asking them to advise us if their contact details change; providing each family with an Aus $20 gift voucher for a popular grocery store chain at each data collection point and sending thank you notes; and employing project offices who have strengths in the building of rapport with families. The decision to employ these strategies was primarily based on the positive experiences of the research team in the previous research projects [34,35].

A total of 80 volunteers and 80 comparison group members will be recruited to the study, reflecting current volunteer numbers.

#### Participant Timeline

Data collection spans a 15-month period so that there is at least one data collection point post family exit from the Volunteer Family Connect program (families receive the program for 3-12 months depending on their support needs). Volunteer outcomes will be measured over a 12-month period at 6-month intervals.

### Intervention

#### Development

The Volunteer Family Connect program was developed in conjunction with 3 leading not-for-profit organizations, all involved in coordinating volunteer home visiting programs in the eastern states of Australia: The Benevolent Society; Good Beginnings Australia (later subsumed into Save the Children Australia); and Karitane. An executive member from each partner organization along with the research leaders met every 6 weeks for approximately 5 years. Collaboratively, a “best practice” model of volunteer home visiting was developed based on a shared theory of change, the strengths of the existing programs, practice wisdom, and existing research evidence. The program was manualized, and practice tools were created such as fidelity checklists, family progress tools, and volunteer training schedules. The Volunteer Family Connect program is currently being implemented with fidelity in 7 trial sites across 4 states (New South Wales, Queensland, Tasmania, and Victoria) including city, suburban, and rural settings.

Preparation for the trial has also involved extensive and ongoing consultation and support for program coordinators and provision of training across all levels of the partner organizations (including volunteers) to increase the understanding of, and support for, the conduct of an RCT.

#### Pilot Study

Early preparatory work included a pilot and feasibility study. The methods and results of this small study comparing Volunteer Family Connect families with supported playgroup parents over a 6-month period have been reported elsewhere [36]. Family outcome measurement tools were piloted for face validity and ease of use, and the project survey instrument was refined in line with parent feedback and researcher experience of administration. The range of “usual care” programs and services (ie, programs and services available to all members of the community) were identified, and the processes for family recruitment to the trial and randomization were established and tested.

#### Delivery in the Trial

The families assigned to the intervention group will receive the Volunteer Family Connect program delivered by a volunteer associated with one of the partner organizations in the 7 trial sites. The Volunteer Family Connect program comprises the core components described below.

1. Program Coordinators: Each site has an employed program coordinator with tertiary qualifications in social work or a related field. The program coordinators are responsible for recruiting and training the volunteers, establishing referral networks, matching volunteers with families, providing regular supervision to volunteers, conducting intake and progress interviews with families, and referring families to other services within the community.
2. Trained volunteers: All community volunteers participate in a minimum of 30 hours of training before being matched with a family and must participate in two additional capacity-building sessions each year. Examples of core training modules include “a strengths-based approach to working with families,” “reflection on personal values and attitudes,” “boundaries and self-care,” “child development,” and “community resources.” The topics for ongoing capacity-building sessions are decided by the program coordinator depending on family needs at the time. For example, if there are high numbers of families who have infants, topics like “breastfeeding” or “sleeping and settling” may be chosen. All volunteers undergo a background check.
3. Matching: Program coordinators match families with a volunteer, guided by the needs of the family but limited by the pool of volunteers available.
4. Home visits: Volunteers visit the family for approximately 2 hours every week. What happens during visits will depend on the needs of the family. Volunteers are encouraged to support families to connect with other services and facilities within the community (eg, attend local playgroups, visit the child and family health center, go to the park, etc) and link them with information as needed. Volunteers are also expected to model positive interactions with the children and encourage the parents in their personal and family goals. Volunteers do not do cooking or housework tasks unless it is with the parent as part of helping them to learn how to do these tasks, and they do not provide child-minding or child care such as changing nappies or bathing children. Volunteers complete checklists following each home visit, detailing the activities and topics of discussion with the family and whether information was provided to the family or the family linked with another service in the community. The collated data are used as a measure of program fidelity and provide ongoing quality feedback to the service partner organizations.
5. Exit interviews: The duration of the service will be a minimum of 3 months and a maximum of 12 months. When the family, volunteer, and program coordinator agree that the family has met their goals, the family is exited from the program and referred to other services by the program coordinator as appropriate.

#### Control Group, Services as Usual

Neither intervention nor control group families are limited in the extent to which they are able to access other services within the community. It is anticipated that most families will access a range of early childhood health and education services. Family use of other services will be documented in the research, based on parent self-report, and explored as a variable in analysis.

**Table 1:** Outcome measures.

Outcomes and Construct Measured			Data Collection Schedule						Instrument
			Enrollment (Baseline)	Months					
				3	6	9	12	15	
**Primary Outcomes**									
	Community connectedness		✓	✓	✓	✓	✓	✓	Community Connectedness Scale [37]; Social Provisions Scale [38].
	Parenting competence		✓	✓	✓	✓	✓	✓	Parenting Sense of Competence Scale [39].
**Secondary Outcomes**									
	**Parent**								
		Parent physical and mental health	✓	✓	✓	✓	✓	✓	SF-12^a^ [40].
		General parent well-being	✓	✓	✓	✓	✓	✓	The Outcome Rating Scale [41].
		Parent empowerment	✓	✓	✓	✓	✓	✓	Modified Patient Enablement Instrument [42].
		Child-parent relationship	✓	✓	✓	✓	✓	✓	Parental questionnaire (questions from the Canadian National Survey of Parents of Young Children) [43].
	**Child**								
		Immunization	✓	✓	✓	✓	✓	✓	Child Personal Health Record.
		Nutrition or breastfeeding	✓	✓	✓	✓	✓	✓	Parental questionnaire-Breastfeeding questions from the New South Wales Child Health Survey (CHS; CHS items CBF^b^1, CBF2, and CBF13) [44].
		Accidental injury	✓	✓	✓	✓	✓	✓	Parental questionnaire: “In the last 3 months, did your children get injured at home? If yes, did the injury require medical attention (eg, your child needed to go to a hospital emergency room or general physician for medical attention)?”
	**Family**								
		Sustainability of family routines	✓	✓	✓	✓	✓	✓	Ecocultural Family Interview [45].
	**Service use**								
		Satisfaction with the VFC^c^ program and other services being accessed	✓	✓	✓	✓	✓	✓	Checklist of local community services. Modified Patient Satisfaction Questionnaire Short Form [46]. Rating of Expectations identified at program entry on a 10-point Likert Scale *(intervention only).* Semistructured interview at program exit to reflect on experience of program participation *(intervention only)*.
	**Volunteer**								
		Volunteer mental and physical health	✓		✓		✓		SF-12 [40].
		Guidance, reassurance of worth, social integration, nurturance, reliable alliances, and attachment	✓		✓		✓		Social Provisions Scale [38].
		Social connectedness and knowledge of community resources	✓		✓		✓		Community Connectedness Scale [37].
		Confidence or belief in meaningfulness of volunteer participation	✓		✓		✓		Community Service Self-Efficacy Scale [47,48].
		Motivational drives of volunteers	✓		✓		✓		Volunteer Motivation Inventory [49].
	**Process**								
		Volunteer activities or Experience of program participation		✓^d^	✓^d^	✓^d^	✓^d^	✓^d^	Visit record sheet checklist is completed at every visit. Volunteer visits weekly for a minimum of 3 mo and a maximum of 12 mo. Volunteer completes a checklist of topics and activities covered during the visit and qualitative questions, including volunteer perception of the impact of the program on the family.

^a^SF-12: 12-Item Short Form Health Survey.

^b^CBF: child breastfeeding.

^c^VFC: Volunteer Family Connect.

^d^Collected weekly for the duration of the intervention.

### Outcomes

In keeping with the processes of a pragmatic randomized trial [37], primary and secondary outcomes were chosen in collaboration with the partner organizations and in consultation with volunteers and families, based on their perceptions of the expected benefits of volunteer home visiting and the importance of the outcome to the families and their volunteers. Discussions on appropriate outcomes were conducted in monthly steering committee meetings with senior representatives from all partner organizations, in focus groups with volunteers held in every participating site, and in focus groups with families conducted within the Sydney-based sites. Wherever possible, tools previously demonstrated to have power to show significant differences between the intervention and comparison groups with a minimum of 100 participants per group were selected; however, many of the expected outcomes have not previously been measured in home visiting studies. With the exception of the home visiting program satisfaction scale (intervention group only), measures are identical for both family intervention and comparison groups. Measures are identical for the volunteer group and the matched community comparison group. In addition, program process data will be collected. The measures are presented in Table 1.

### Allocation

Most families will be allocated on an individual basis using computer-generated randomizing, giving them an equal chance of being allocated to the intervention or the control group. If more families are recruited than the number of available volunteers, randomization will be proportional using computer-generated randomizing (eg, if there are five available volunteers and seven recruited families, five of the seven families will be randomly allocated to the intervention group and two to the usual care group).

Family group random allocation will be the responsibility of the research program manager who will be blind to any details about the family when making this allocation. Once the allocation is determined, the program manager will notify the appropriate program coordinator. It will not be possible to blind the research staff responsible for data collection: families will know their allocation and are likely to disclose this to the researchers during data collection. While data collection is not blind, data analysis will be blind, completed by team members who have not been involved in data collection.

### Data Collection, Management, and Security

Interviewers are trained in the standard administration of the instruments and handling of distressed parents or volunteers. The research team meets at least monthly to review interview techniques and ensure consistency of administration. All data are checked to ensure accuracy and consistency of data entry.

Family participants will complete a survey every 3 months for 15 months, commencing at recruitment and continuing until 15 months post their own recruitment date. The baseline and follow-up surveys will be collected by a research assistant (at the home of the participant or over the phone) or self-completed by participants if preferred. Surveys can be completed on a paper form or a word document sent via email, or they can be Web based using Qualtrics software (Qualtrics, Provo, UT). The Web-based version of the survey has been tested for usability and technical issues in one of the study sites and will eventually be rolled out to all sites. Previous research suggests that offering multiple survey response modes allows participants to choose what is most convenient for them, with little negative impact on data quality [50]. The use of iPads and Web-based survey software has been shown in the previous research to increase efficiency and reliability and to reduce data entry errors [51-53]. All data collected via paper or emailed word documents will be entered into the Web-based survey by a research assistant. Data will be stored in a password-protected Qualtrics database and backed up to a password-protected folder on a server. Only members of the research team will have access to the data.

The Web-based survey will be administered in two sections. Section A includes prefilled items (eg, demographic questions, breastfeeding status, service expectations) for the participant to update (if applicable), and Section B contains all other items. Section A requires a link to be manually generated for each participant at each time point. Section B uses a generic link to the respective time point. For participants self-completing, both Section A and Section B links will be manually emailed by research assistants in each site and reminders (emails, text messages, or phone calls) will be sent weekly until the survey is completed or for 6 weeks post the due date for the survey. Both sections require the participant to enter a unique identifying number at the start of the survey. Where duplicate entries occur, the earliest completed response will be retained.

Items will always be presented in the same order, and adaptive questioning will be used to only display relevant questions to participants. Dependent on adaptive questioning, Section A has a minimum of twelve pages and a maximum of forty-five pages, with a maximum of eight questions per page. Dependent on adaptive questioning, Section B has a minimum of forty-three pages and a maximum of seventy-eight pages, with a maximum of six questions per page. Multiple-choice questions use forced choice validation, with an option of “refused” on all questions. Open-field responses use requested response validation, with a prompt appearing before the survey can be progressed to the next page. A back button will be available to the respondents; however, there is no provision to review the completed survey before submission.

Volunteers and comparison group members can opt to complete their surveys over the telephone with a research assistant who enters their response into the Web-based survey using an iPad, independently using a paper survey that is mailed to them, or Web based using Qualtrics.

All data are stored on password-protected computers at Macquarie University and at Western Sydney University, to which only the research team has access. Data are de-identified during data entry, at which time all names are replaced with participant numbers. Data are stored in accordance with the requirements of the Australian National Health and Medical Research Council and the Privacy Act 1988.

### Data Analysis

#### Quantitative Analysis

Primary and secondary outcomes will be extracted and analyses conducted using SPSS Version 25.0. Analysis will be completed both on an intention-to-treat and a per-protocol basis. Families will be considered to have received the scheduled dose if they receive visits from the volunteer for at least 3 months with no gap between visits of more than 2 weeks. Volunteer outcomes will be analyzed using cross-sectional comparative analysis. Participant demographic data will be analyzed using basic descriptive statistics. Prior to the analysis of outcome measures, data will be assessed for outliers and normality. Scale variables will be analyzed using independent *t* test or analysis of variance or their nonparametric counterparts (eg, Mann–Whitney U test) if appropriate. Mixed modeling will be completed on the primary and secondary family outcomes to assess the effect of the intervention over time while adjusting for possible confounders. Categorical variables will be analyzed using odd ratios or chi-square analysis. For all analyses, two-tailed tests will be undertaken: findings with α<0.05 will be determined to be statistically significant. ESs will be calculated for all trends (α<0.1) and statistically significant findings; (ES≈0.5 [Cohen *d*]) will be considered clinically meaningful. Overall, the program will be considered to have been effective if at least one of the primary outcomes is positive and the other is neutral.

#### Qualitative Analysis

The survey instruments include some open-ended questions. The qualitative data will be extracted into a text file for analysis and entered into NVivo (QSR International). Analysis of the open-ended responses will employ a thematic approach, with themes and relationships between themes identified and described. The first ten interviews will be dual-coded, followed by the development of a coding framework then independent coding with regular checks for inter-rater reliability. Analysis will initially focus on the family as a case and explore change in the family’s reported experience over time. It will then expand to compare themes across the families as a group to capture the collective experience.

### Ethics

Ethics approval for the study was granted by the Macquarie University Human Research Ethics Committee (Reference number: 5201401144).

### Data Availability

The data that support the findings of this study will be made publicly available at the conclusion of the research on request to the corresponding author RG.

## Results

Seed funding for this project was provided by a private philanthropist, who went on to fund the effectiveness trial described here commencing in 2016. Data collection is currently underway and will be complete by the end of 2018. The first results are expected to be submitted for publication early in 2019.

## Discussion

In this study, we aim to explore the effectiveness of a volunteer home visiting program designed to provide support to families with young children who might otherwise “fall between the cracks” because they are not eligible for intensive family support services but need more support than is available through universal primary services. The Volunteer Family Connect study will provide evidence of the outcomes for families based on the program logic of volunteer home visiting [1] and outcomes desired and valued by parents. This study assesses the effectiveness of volunteer home visiting on its own merits and contribution to the service landscape, rather than as a program equivalent to, or potential substitute for, professional services.

RCTs can be an uncomfortable methodological approach for not-for-profit organizations. Their employees are generally guided by an altruistic and empathic approach, rather than a rigidly scientific approach, and randomly denying support to someone they believe would benefit can be challenging. Time was spent with those delivering the program to discuss the ethical situation in the context of delivering the previously untested service that did not have evidence of effectiveness. The lengthy lead in time for this project was essential to secure support for the research across all levels of the organizations, from CEOs and board members, through to program coordinators and volunteers. Some volunteers and program coordinators did not want to be involved, and the services have also experienced some difficulties with their referral networks, with some referrers ceasing to refer families during the research trial. Understanding these significant challenges for participating organizations and their ongoing commitment to ensure a rigorous research approach is commendable. Launching this research is an indication that within the not-for-profit sector, rigorous research is feasible. It does, however, need to be embedded within trusting relationships and will need many formal and informal conversations across all levels of the organization, which can take several years.

Future research will explore different modes of program delivery, including whether volunteer home visiting can be effectively delivered using technology such as telephone and videolinks. This work will complement the existing research exploring the role of technology in providing professional and other support services to those in rural and remote regions [31].

A strength of this study is that it is part of a comprehensive program of research that employs an ecological approach [4] guided by an understanding that the health and well-being of parents, the stability of families, the strength of social interactions, the safety of neighborhoods, and respect for cultural contexts are all equally important for achieving positive outcomes for children. In conjunction with the RCT of family outcomes are the following: (1) a matched comparison study on the benefits of volunteering; (2) a mixed-methods study of program implementation that explores program quality and fidelity; and (3) a social return on investment analysis. This RCT and the three additional studies will interlink and directly inform the analysis and interpretation of findings across the program of research. As a whole, this program of research has the potential to make a significant contribution to our understanding of the support and service needs of vulnerable families and the value of volunteering as a mechanism to mobilize and strengthen communities.

## Abbreviations

ES: effect size
PRECIS: PRagmatic Explanatory Continuum Indicator Summary
RCT: randomized controlled trial
